# Fully automated film mounting in dental radiography: a deep learning model

**DOI:** 10.1186/s12880-023-01064-9

**Published:** 2023-08-18

**Authors:** Yu-Chun Lin, Meng-Chi Chen, Cheng-Hsueh Chen, Mu-Hsiung Chen, Kang-Yi Liu, Cheng-Chun Chang

**Affiliations:** 1 Department of Medical Imaging and Intervention, Chang Gung Memorial Hospital at Linkou, Taoyuan, Taiwan; 2 Department of Medical Imaging and Radiological Sciences, Chang Gung University, Taoyuan, Taiwan; 3 Department of Dentistry, Chang Gung Memorial Hospital at Taipei, Taipei, Taiwan; 4 Department of Dentistry, National Taiwan University Hospital, Hsin-Chu Branch, Hsin-Chu, Taiwan; 5 Department of Dentistry, National Taiwan University Hospital, Taipei, Taiwan; 6 Department of Electrical Engineering, National Taipei University of Technology, 1, Sec. 3, Zhongxiao E. Rd, Taipei, 10608 Taiwan

**Keywords:** Radiography, Dental, Deep learning

## Abstract

**Background:**

Dental film mounting is an essential but time-consuming task in dental radiography, with manual methods often prone to errors. This study aims to develop a deep learning (DL) model for accurate automated classification and mounting of both intraoral and extraoral dental radiography.

**Method:**

The present study employed a total of 22,334 intraoral images and 1,035 extraoral images to train the model. The performance of the model was tested on an independent internal dataset and two external datasets from different institutes. Images were categorized into 32 tooth areas. The VGG-16, ResNet-18, and ResNet-101 architectures were used for pretraining, with the ResNet-101 ultimately being chosen as the final trained model. The model’s performance was evaluated using metrics of accuracy, precision, recall, and F1 score. Additionally, we evaluated the influence of misalignment on the model’s accuracy and time efficiency.

**Results:**

The ResNet-101 model outperformed VGG-16 and ResNet-18 models, achieving the highest accuracy of 0.976, precision of 0.969, recall of 0.984, and F1-score of 0.977 (p < 0.05). For intraoral images, the overall accuracy remained consistent across both internal and external datasets, ranging from 0.963 to 0.972, without significant differences (p = 0.348). For extraoral images, the accuracy consistently achieved the highest value of 1 across all institutes. The model’s accuracy decreased as the tilt angle of the X-ray film increased. The model achieved the highest accuracy of 0.981 with correctly aligned films, while the lowest accuracy of 0.937 was observed for films exhibiting severe misalignment of ± 15° (p < 0.001). The average time required for the tasks of image rotation and classification for each image was 0.17 s, which was significantly faster than that of the manual process, which required 1.2 s (p < 0.001).

**Conclusion:**

This study demonstrated the potential of DL-based models in automating dental film mounting with high accuracy and efficiency. The proper alignment of X-ray films is crucial for accurate classification by the model.

## Introduction

Dental radiography, a crucial diagnostic tool in dentistry, provides detailed images of the teeth, jaw, and surrounding structures [1]. Both intraoral and extraoral radiographic images are instrumental in dental practice. While intraoral images primarily focus on individual teeth or small groups of teeth, extraoral images capture a comprehensive view of the larger anatomical structures in the maxillofacial region, including the jaws, temporomandibular joints, sinuses, and other adjacent structures [2].

The accurate interpretation of dental X-rays requires the proper alignment and positioning of the film, which is generally conducted manually by dental radiographers or dentists [3]. This process involves rotating and identifying the correct position of the film and placing it in the appropriate tooth area. Because this process relies on the subjective judgement of the radiographer, it is time-consuming and prone to error [4].

In recent years, deep learning (DL) has emerged as a powerful tool for automating various image-related processes, such as identification and classification [5–7]. Studies have established the ability of DL models to analyze complex patterns and relationships in data, thereby advancing the accuracy and efficiency of various processes [8] and providing a promising solution for enhancing the interpretation process in dental radiography [9]. For instance, Lee et al. [10] and Bayrakdar et al. [11] leveraged the potential of convolutional neural network (CNN) algorithms for the detection and diagnosis of dental caries. Murata et al. [12] employed a CNN for the evaluation of maxillary sinusitis on panoramic radiography. Despite these advancements, the field has yet to explore one critical area — automated film mounting in dental radiography. This process, encompassing both intraoral and extraoral images, holds the potential to significantly enhance X-ray interpretation. By automating the time-consuming and error-prone task of manual film mounting, clinicians can focus on X-ray interpretation, resulting in increased accuracy and efficiency. To our knowledge, this study is the first to propose an automatic film mounting method for dental X-rays.

In this study, we developed and assessed a DL model for the accurate automated identification, rotation, and mounting of both dental intraoral and extraoral films. The goal is to enhance the efficiency and accuracy of the dental radiography interpretation process.

## Methods

### Patients and datasets

The Institutional Review Board of Chang Gung Medical Foundation approved this study (IRB number: 201900816B0C501), and also granted a waiver for the requirement of written informed consent. This study retrospectively enrolled a total of 1,500 patients at the Taipei branch of CGMH from July 2019 to June 2021 to train the model. The training dataset comprised a total of 23,379 images, including 22,344 intraoral images and 1,035 extraoral images. To enhance the diversity of the dataset, the training data were augmented four times by rotating the films to 0°, 90°, 180°, and 270°. The data were divided into training and validation sets using 5-fold cross-validation to prevent overfitting. An additional 2,333 independent images were employed to test the model’s performance; these included 2,221 intraoral and 112 extraoral images (Table 1).

**Table 1:** Number of images in training and testing datasets

Institution	Internal dataset Taipei CGMH	External dataset	
		Linkou	Taoyuan
Training Data	23,379	-	-
Intraoral	22,344 (95.6%)	-	-
Extraoral	1035 (4.4%)	-	-
Testing Data	2333	1916	1645
Intraoral	2221 (95.2%)	1828 (95.3%)	1565 (95.1%)
Extraoral	112 (4.8%)	88 (4.6%)	80 (4.9%)

To test the model’s generalization capabilities, external testing was performed using independent datasets obtained from two additional hospitals. The first hospital, the Linkou branch of CGMH, provided 1,828 intraoral images and 88 extraoral images. The second hospital, the Taoyuan branch of CGMH, supplied 1,565 intraoral images and 80 extraoral images. These images were not included in the training phase of the DL model, thereby offering a more rigorous test of the model’s ability to generalize to unseen data, a characteristic critical for real-world applications.

### Data labelling

The matrix of intraoral images included Dental CR#0 (380 × 400 pixels), Dental DR#2 (800 × 800 pixels), Dental CR#2 (550 × 700 pixels), and Dental CR#4 (1100 × 800 pixels). The matrix of extraoral images included Panorex (1200 × 800 pixels), temporomandibular joint (TMJ; 1200 × 2400 pixels), and cephalometric (1500 × 3000 pixels) images.

We collected intraoral films with corresponding labels for the correct tooth position for each film. We categorized the data into 32 dental regions in accordance with the standard positioning guidelines for dental radiography, comprising 28 intraoral and 4 extraoral categories. The intraoral images included 14 categories of periapical images, 2 categories of bitewing (BW) images, 4 categories of vertical BW (VBW) images, 2 categories of occlusal images, 2 categories of pediatric upper (52–62) and lower (72–82) arch images, and 4 categories of pediatric upper (53 − 16, 63 − 26) and lower (73 − 36, 83 − 46) images. The extraoral images included one category of Panorex images, one category of TMJ images, and two categories of cephalometric (posterior–anterior and lateral) images. The criteria used to categorise the images are detailed in Table 2.

**Table 2:** **Categorisation criteria of intraoral images**

Teeth area	Intraoral film direction	Image selection criteria
Upper and lower teeth 2 ~ 2	Straight, Horizontal	Image contains upper and lower center incisors and lateral incisors, a few contain children’s upper and lower incisors.
Upper and lower teeth 3 ~ 5	Straight	Image contains upper and lower canines, first premolars and second premolars.
Upper and lower teeth 4 ~ 6	Straight, Horizontal	Image contains upper and lower first premolars, second premolars and first molars.
Upper and lower teeth 6 ~ 8	Straight, Horizontal	Image contains the upper and lower first molars, second molars and the third molars (wisdom teeth).
BW: Left and right sides	Horizontal	The adult image includes the upper and lower first premolars, second premolars, first molars and the second molars. The child image includes the upper and lower canines(3), first molars(4) and the second molars(5) .
VBW Left and right front	Straight	The adult image includes the upper and lower first premolars, second premolars and a few of the first molars. The child image includes the upper and lower first molars(4) and second molars(5).
VBW Left and right behind	Straight	Image contains the upper and lower first molars and second molars; a few contain second premolars.
Occlusal Upper and lower	Straight, Horizontal	Image contains adult and children Upper and lower front teeth.
Pediatric upper (52 ~ 62) and lower (72 ~ 82) arch images	Straight, Horizontal	Image contains upper and lower center incisors and lateral incisors. A few contain adult upper and lower center incisors and lateral incisors.
Pediatric upper (53 ~ 16, 63 ~ 26) and lower (73 ~ 36, 83 ~ 46) images	Straight, Horizontal	The image includes upper and lower canines(3), first molars(4) and the second molars(5). A few include adult upper and lower canines, first premolars, second premolars and the first molars(6).

### Network training

We initially trained the DL models using three different networks: VGG-16, ResNet-18, and ResNet-101 [13]. After comparing their preliminary accuracies, we adopted ResNet-101 as the final model due to its superior performance. During the training process, we implemented the Adam optimization algorithm and the categorical cross-entropy loss function. Other hyperparameters included the following: number of epochs = 100; learning rate = 0.1; batch size = 32; and weight decay = 0.001.

The network was trained on an Intel Xeon E5-2650 with 16GB DRAM, using a GTX-1080 GPU. The software, which was written in Python 3.5.4, used Keras 2.1.4 and TensorFlow 1.5.0.

After categorizing the images, a visualization tool was developed that automatically oriented and positioned the films on a standard template. The template, a standardized grid or reference image, helped align the films to their correct positions, ensuring a consistent and uniform presentation of the radiographs.

### Workflow of the DL model inference

**Fig. 1:**
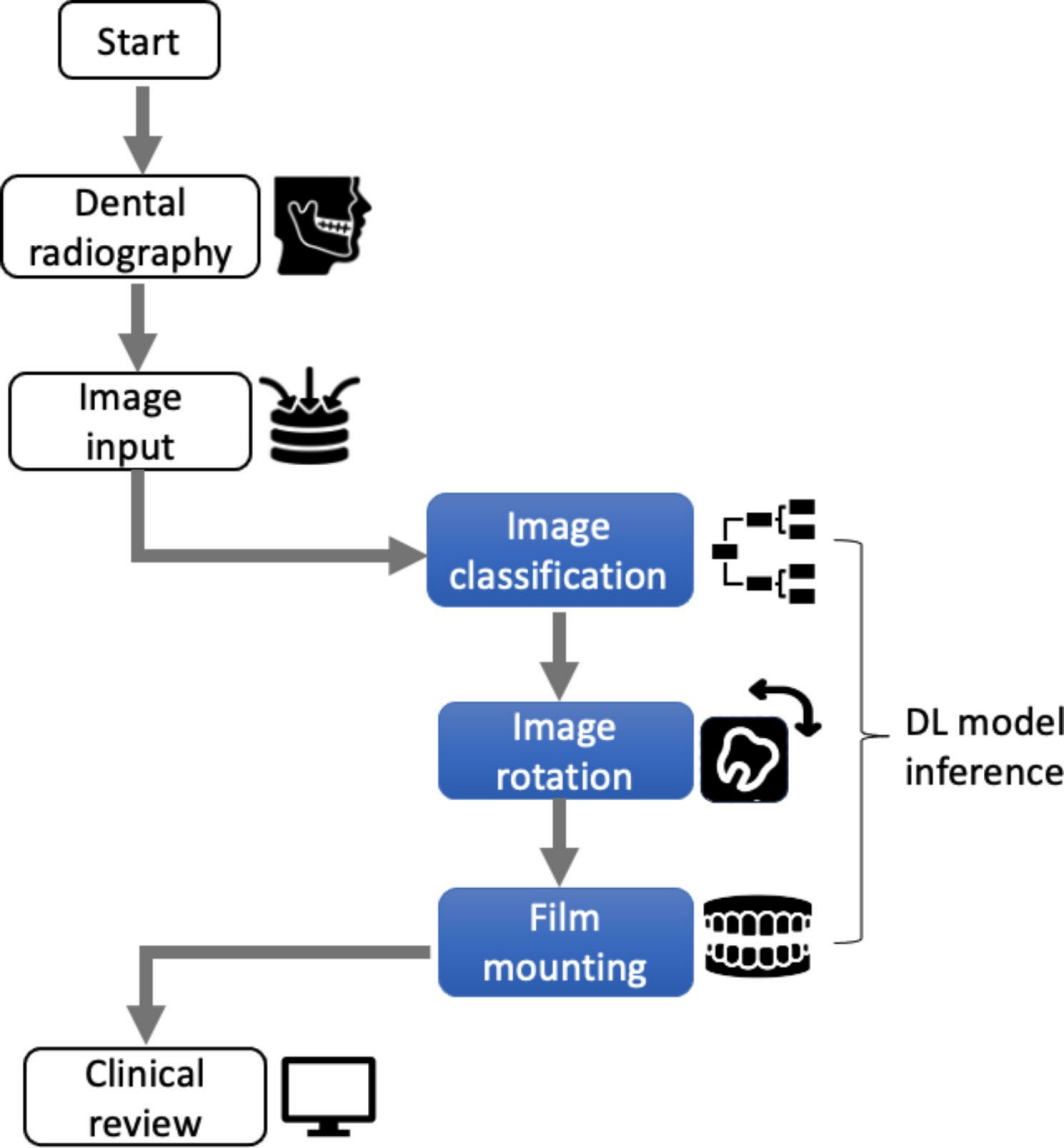
Workflow of the deep learning (DL) tool for automated dental film mounting The workflow begins with the process initiation (“Start”) and patient undergoing dental radiography where both intraoral and extraoral images are captured (“Patient Radiography”). These raw images are then fed into the DL tool (“Image Input”). The model classifies each image into a specific tooth area (“Image Classification”), and subsequently, these images are automatically rotated to their correct orientation (“Image Rotation”). Following rotation, the images are digitally mounted in the correct orientation, which mimics traditional physical film mounting, thereby providing a comprehensive view of the dental structures (“Film Mounting”). Finally, the mounted images are presented to the clinician for review and interpretation (“Clinician Review”). The steps highlighted in blue represent the process of DL model inference

Figure 1 presents the workflow of our proposed DL model tool. This process begins with a patient undergoing dental radiography, yielding intraoral or extraoral images. The DL model takes these raw images as input, identifying and classifying each one into a specific tooth area. Subsequent automatic rotation ensures all images align correctly. The tool then executes digital film mounting, arranging the images in their appropriate positions to create a holistic view of the dental structures, echoing the conventional physical film mounting process. The final product is a set of well-organized, mounted images ready for clinical review and interpretation.

### Evaluation of misalignment

To assess the effect of film misalignment on the performance of the DL model, we conducted experiments using a real human dental skull model by tilting the films at various angles relative to the X-ray tube. The tilting angles ranged from − 15° to + 15°, with increments of − 10°, − 5°, 0°, 5°, and 10°, with 0° indicating perfect alignment between the film and the X-ray tube. Three intraoral films were obtained for each angle and tooth position. This process aimed to evaluate the model’s ability to detect subtle changes in image orientation caused by film tilt and its effect on the accuracy of tooth position recognition.

### Performance evaluation

We utilized the trained model to classify the test images into 32 tooth position classes and aligned the radiographs based on the predicted tooth positions. By comparing the results with the actual labels, we were able to determine the performance of the trained DL model. The performances were assessed for each fold in the five-fold cross-validation, and the average across these folds was deemed the final performance of the DL model for each network. The performance evaluation metrics employed for the models included (1) Accuracy, (2) Precision, (3) Recall, (4) F1 score.

1 $$Accuracy= \frac{TP+TN}{TP+TN+FP+FN}$$

2 $$Precision= \frac{TP}{TP+FP}$$

3 $$Recall= \frac{TP}{TP+FN}$$

4 $$F1 score=2\times \frac{Recall\times Precision}{Recall+Precision}$$

Where TP, FP, FN and TN represent true positive, false positive, false negative, and true negative, respectively.

### Time of tasks

To assess the efficiency of the DL model, we calculated the time required for the model to complete the tasks of image identification, rotation, and mounting. To provide a comparative context, we randomly selected a sample of 50 patients, and measured the time duration consumed in performing the same tasks manually. This facilitated a direct comparison of the time efficiencies between the manual process and the DL model’s operation.

### Statistics

Statistical analysis was performed using GraphPad Prism (version 8.0). We employed descriptive statistics to summarise the data and determine the mean accuracy, standard deviation, and range of scores. The variations in accuracy across different hospitals and among the distinct tilt angle groups were examined using an analysis of variance (ANOVA) test. To assess efficiency, we employed Student’s t-test to compare the time required by manual processing with that of the trained model. Statistical significance was indicated at p < 0.05.

## Results

### Model performance

This study employed a total of 5,894 images to test the performance of the trained model, which included 5,614 intraoral images and 280 extraoral images from 3 institutes (Table 1). Table 3 presents the classification performance of the pre-trained DL models on the internal test dataset. The ResNet-101 model demonstrated superior performance with the highest accuracy of 0.976 (95% CI: 0.968–0.983), precision of 0.969 (95% CI: 0.951–0.981), recall of 0.984 (95% CI: 0.969–0.991), and F1-score of 0.977 (95% CI: 0.969–0.984). These results significantly outperformed the performances of the VGG-16 and ResNet-18 models (p < 0.05 for all).

**Table 3:** Performances of the pre-trained DL models on the internal test dataset

Network	Accuracy	Precision	Recall	F1-Score
VGG-16	0.934 (0.912, 0.957)	0.916 (0.895, 0.932)	0.956 (0.933, 0.973)	0.937 (0.917, 0.959)
ResNet-18	0.963 (0.948, 0.978)	0.952 (0.941, 0.963)	0.977 (0.962, 0.989)	0.965 (0.951, 0.979)
ResNet-101	0.976 (0.968, 0.983)	0.969 (0.951, 0.981)	0.984 (0.969, 0.991)	0.977 (0.969, 0.984)

Data are presented as means with 95% confidence intervals

Table 4 displays the accuracies of ResNet-101 model’s image classification for each tooth position on both internal and external test datasets. For intraoral images, the overall accuracy was 0.972 (95% CI: 0.965–0.98) for Taipei CGMH, 0.963 (95% CI: 0.955–0.972) for Linkou CGMH, and 0.967 (95% CI: 0.961–0.974) for Taoyuan CGMH. The differences were not statistically significant (p = 0.348). For extraoral images, the accuracy consistently achieved the highest value of 1 across all institutes.

**Table 4:** Accuracy of image classification of ResNet-101 model for each tooth position

Tooth position	Accuracy		
	Internal testing Taipei CGMH	External testing	
		Linkou	Taoyuan
16 ~ 18	0.954	0.946	0.961
16 ~ 14	0.942	0.932	0.941
13 ~ 15	0.950	0.934	0.945
12 ~ 22	0.972	0.952	0.962
23 ~ 25	0.962	0.958	0.956
24 ~ 26	0.984	0.975	0.971
26 ~ 28	1.000	0.986	0.975
46 ~ 48	0.992	1.000	0.986
46 ~ 44	0.946	0.938	0.952
43 ~ 45	0.979	0.962	0.963
42 ~ 32	0.983	0.952	0.964
33 ~ 35	0.963	0.952	0.973
34 ~ 36	0.972	0.983	0.971
36 ~ 38	0.976	0.962	0.973
Occlusal Upper	1.000	1.000	1.000
Occlusal Lower	1.000	1.000	1.000
53 ~ 16	0.946	0.933	0.958
52 ~ 62	0.992	0.982	0.985
63 ~ 26	0.968	0.963	0.965
83 ~ 46	0.984	0.956	0.978
72 ~ 82	1.000	0.976	0.985
73 ~ 36	0.978	0.964	0.963
BW-Right	0.992	0.994	0.986
BW-Left	0.975	0.986	0.971
VBW-Right anterior	0.967	0.952	0.946
VBW-Right posterior	0.976	0.963	0.966
VBW-Left anterior	0.942	0.952	0.943
VBW-Left posterior	0.934	0.921	0.938
Panorex	1.000	1.000	1.000
TMJ	1.000	1.000	1.000
Cephalometric posterior-anterior	1.000	1.000	1.000
Cephalometric Lateral	1.000	1.000	1.000

The accuracy among the three hospitals did not differ, with accuracies of 0.976 (95% CI: 0.969–0.983) for Taipei CGMH, 0.968 (95% CI: 0.959–0.977) for Linkou CGMH, and 0.971 (95% CI: 0.964–0.978) for Taoyuan CGMH (p = 0.348).

### Influence of alignment tilt angles on model accuracy

**Fig. 2:**
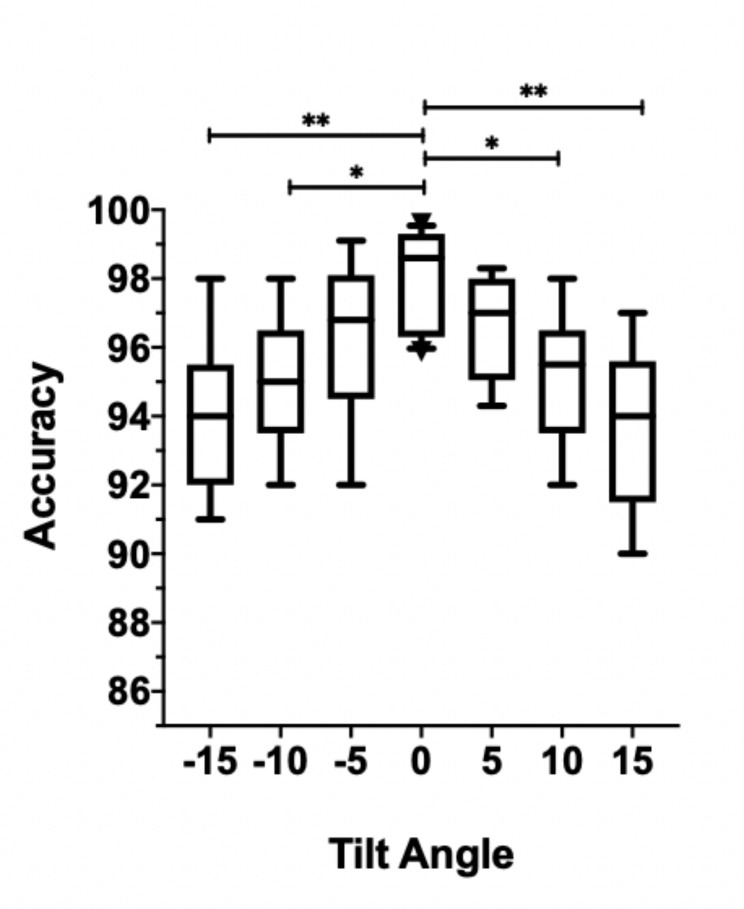
Influence of film tilt angles versus model’s accuracy

Figure 2 illustrates the effect of film tilt angles on the accuracy of the model. The tilt angle of X-ray films affected the model’s accuracy. The control group, with X-ray films correctly aligned at 0°, achieved an accuracy of 0.981 (95% CI: 0.968–0.991). X-ray films with a slight misalignment of ± 5° achieved an accuracy of 0.964 (95% CI: 0.953–0.975, p = 0.02 compared with the control group), whereas those with a moderate misalignment of ± 10° achieved an accuracy of 0.95 (95% CI: 0.936–0.971, p < 0.05 compared with the control group). The group with a severe misalignment of ± 15° had the worst performance, achieving an accuracy of 0.937 (95% CI: 0.918–0.956, p < 0.001 compared with the control group).

**Fig. 3:**
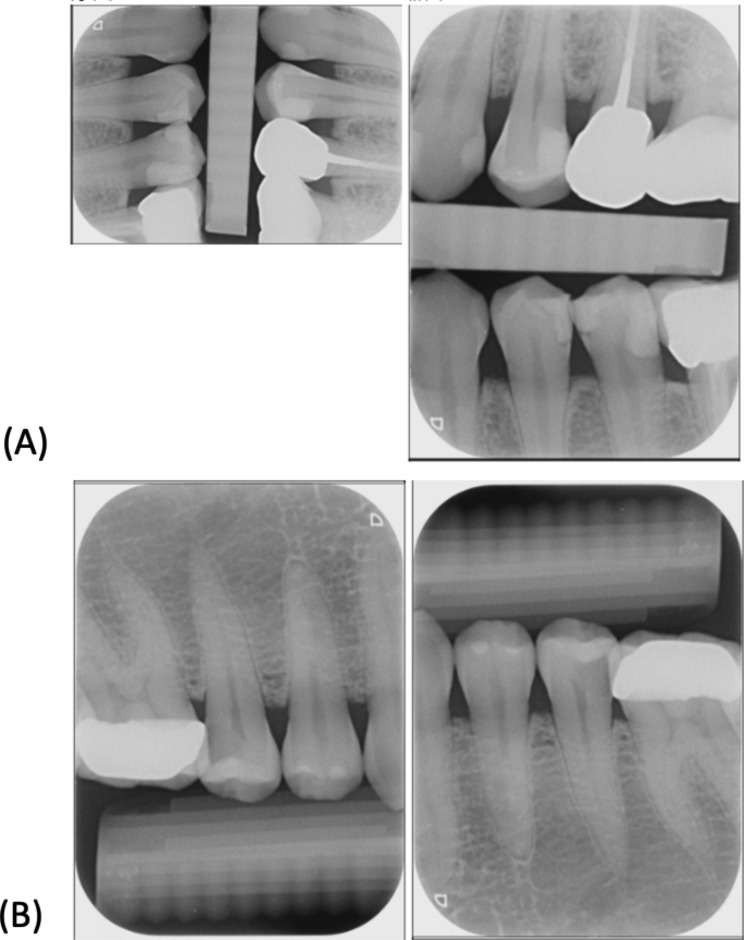
Examples of intraoral films that were correctly rotated and classified by the DL model. The left column of each figure displays the original image, and the right column displays the corrected image as processed by the model. (**A**) The original image is a left anterior view of the upper teeth, including the second and third premolars (VBW film). The model correctly rotated and identified the film as a left anterior VBW film with a probability of 0.99972. (**B**) The model correctly rotated and identified the film as the “34–36” position

**Fig. 4:**
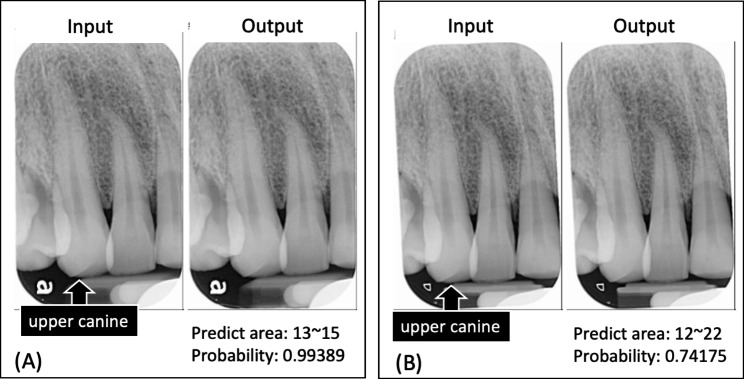
Example of an intraoral film that was incorrectly classified by the AI model. (**A**) When the operator takes the X-ray with a horizontal tilt angle of 0 degrees, the AI model correctly recognizes the desired tooth area to be captured. However, when the tube tilts from the medial to distal by 15 degrees (**B**) The projected image resembles the capture angle of teeth 12–22, leading the AI model to categorize it into the 12–22 tooth area

Figures 3 and 4 illustrate the performance of the DL model in automating the dental film mounting process. The DL model is adept at correcting orientations and rotating images to achieve proper alignment. As depicted in Fig. 3, the developed DL model accurately classified and positioned intraoral films, even when misaligned or inverted. Conversely, Fig. 4 presents a case in which the DL model misclassified an intraoral film. This error occurred due to a substantial 15° tilt of the X-ray tube, causing the projected image to resemble a different position and leading the DL model to incorrectly classify the tooth area.

### Time of tasks

The results underscored a substantial enhancement in time efficiency by adopting our DL model in contrast to the traditional manual method. In the testing phase, the DL model adeptly executed image rotation and classification tasks, demonstrating significant time savings (0.17 ± 0.02 s per image for the DL model vs. 1.2 ± 0.28 s manually; p < 0.001). Of particular note, the per-patient processing time further exemplified the efficiency of the model. While manual processing required 118.6 ± 28.5 s per patient, the DL model drastically cut this down to only 3.3 ± 0.41 s (p < 0.001). The DL model’s processing time remained consistent, regardless of the film type, whether BW or periapical (p = 0.125), attesting to its robust performance across diverse imaging modalities.

## Discussion

The results of this study demonstrate the effectiveness of the developed DL model in automating dental film mounting. The model achieved a high accuracy of 97.2% for intraoral images and 100% for extraoral images, demonstrating consistent performance across internal and external institutions without significant differences. These findings suggest that the DL model can serve as a valuable tool in dental practice, streamlining the film mounting process and potentially reducing the risk of misdiagnosis or treatment errors stemming from incorrect film interpretation.

The high accuracy for intraoral images indicates the model effectively recognizes and classifies tooth positions within the oral cavity, which is crucial for accurate dental film mounting [10]. The consistent performance across institutions suggests robustness and generalizability, making it a reliable tool for dental practitioners [11]. The high accuracy for extraoral images demonstrates the model’s ability to differentiate between intraoral and extraoral images, preventing interchange errors during mounting [14]. The notable success of our study lies in our approach to model training and the inherent qualities of the images used. Our DL model was extensively trained on a diverse dataset, allowing it to effectively classify a wide range of unique features. In particular, the high accuracy in classifying extraoral images is attributed to their distinct anatomical landmarks, such as sinuses and nasal bones, which serve as reliable classification indicators. Further, the reduced variability in these images compared to intraoral ones simplifies the task, aiding in the DL model’s superior performance.

The study highlights the impact of tilt angle on the model’s accuracy, emphasizing the importance of proper X-ray film alignment for accurate classification by the DL model. Practitioners should ensure correct alignment during image acquisition to optimize performance. Robustly adapting models to varying film angles is crucial, and including diverse images with different angles in the training dataset could address this issue [15]. However, collecting a comprehensive dataset may be challenging. The DL model accurately classified and mounted most intraoral films in the dataset, suggesting that DL could significantly improve the film mounting process in dental radiography.

Although the model performed well in most cases, it struggled with classification in certain scenarios, such as when the X-ray tube was significantly tilted. This highlights the need for careful consideration when designing and implementing DL systems in clinical practice and emphasizes the importance of selecting and curating the dataset used for training and testing. Despite some limitations, the DL model detected subtle changes in angle deviation and generated results generally acceptable to clinical dentists.

In terms of efficiency, the DL model’s ability to process images with significantly reduced time relative to manual methods underscores its potential in streamlining workflow in dental radiography. While the per-image time savings might seem small, the DL model drastically reduced the per-patient processing time. In a manual setting, operators are required to handle each image individually, taking into account their correct alignment and position. Furthermore, there can often be pauses, hesitations, or fatigue-related slowdowns that occur when operators manually process a series of images. This can lengthen the overall processing time significantly, especially when scaled to a larger number of patients. The minutes saved through the use of our DL model can be reallocated to more critical aspects of patient care such as diagnosis and treatment planning, consequently enhancing overall dental healthcare efficiency.

Our study has limitations. Firstly, our focus was primarily centered on the accuracy of the film mounting process, and we did not explore the diagnostic accuracy of the images processed using the DL model in depth. To confirm its clinical impact, future research must examine how the DL model affects diagnostic accuracy. Secondly, the model’s performance may vary across different dental practices due to the training dataset’s limited diversity. Ensuring its broad clinical applicability requires a more comprehensive dataset, covering various patient demographics, tooth morphologies, clinical conditions, and imaging techniques. Finally, while the model demonstrated high accuracy, there may be instances of minor misalignments due to varied clinical practices. As such, a future avenue of improvement could include the introduction of data augmentation with more subtle rotational degrees, improving the model’s ability to manage minor misalignments and potentially enhancing its robustness.

In conclusion, the results of this study demonstrated the potential of DL in automating the dental film mounting process. The DL model exhibited a high level of accuracy and efficiency in classifying and mounting dental films, which could greatly enhance the workflow in dental radiography. The results also highlighted the importance of proper X-ray film alignment for accurate classification by the DL model.

## Data Availability

The datasets used during the current study are available from the corresponding author on reasonable request.
